# Fecal microbiome as determinant of the effect of diet on colorectal cancer risk: comparison of meat-based versus pesco-vegetarian diets (the MeaTIc study)

**DOI:** 10.1186/s13063-019-3801-x

**Published:** 2019-12-09

**Authors:** Francesco Sofi, Monica Dinu, Giuditta Pagliai, Fabrice Pierre, Francoise Gueraud, Jildau Bowman, Philippe Gerard, Vincenzo Longo, Lisa Giovannelli, Giovanna Caderni, Carlotta de Filippo

**Affiliations:** 1 Department of Experimental and Clinical Medicine, School of Human Health Sciences, University of Florence, Largo Brambilla 3, 50134 Florence, Italy; 2 Unit of Clinical Nutrition, Careggi University Hospital, Florence, Italy; 3 Don Carlo Gnocchi Foundation Italy, Onlus IRCCS, Florence, Italy; 4 INRA, ToxAlim (Research Centre in Food Toxicology), Universitè de Toulouse, ENVT, INP-Purpan, UPS, Toulouse, France; 5 Netherlands Organisation for Applied Scientific Research (TNO) Research group: Microbiology and Systems Biology (MSB), Amsterdam, The Netherlands; 6 Micalis Institute, INRA, AgroParisTech, Université Paris-Saclay, Jouy-en-Josas, France; 7 Institute of Agricultural Biology and Biotechnology (IBBA), National Research Council (CNR), Pisa, Italy; 8 Department of Neuroscience, Psychology, Drug Research and Children’s Health, University of Florence, Florence, Italy

**Keywords:** Diet, Prevention, Neoplastic disease, Meat, Vegetarian, Intestinal microbiome, Colorectal cancer

## Abstract

**Background:**

Convincing evidence suggests that the risk of colorectal cancer (CRC) is increased by the typical Western diet characterized by high consumption of red and processed meat. In addition, some epidemiological studies suggest a reduction in the risk of CRC associated with fish consumption. The role of the gut microbiome in this diet-associated risk is not well understood.

**Methods/design:**

This is a randomized parallel open clinical trial comprising a total of 150 clinically healthy subjects randomly assigned to three groups: a meat-based diet of which 4 portions per week are red meat (1 portion = 150 g), 3 portions per week are processed meat (1 portion = 50 g), and 1 portion per week is poultry (1 portion = 150 g), for a total amount of 900 g per week of meat and derivatives; a meat-based diet supplemented with alpha-tocopherol; and a pesco-vegetarian diet excluding fresh and processed meat and poultry, but which includes 3 portions per week of fish for a total amount of 450 g per week. Each intervention will last 3 months. The three diets will be isocaloric and of three different sizes according to specific energy requirements. Anthropometric measurements, body composition, and blood and fecal samples will be obtained from each participant at the beginning and end of each intervention phase. The measure of the primary outcome will be the change from baseline in DNA damage induced by fecal water using the comet assay in a cellular model. Secondary outcome measures will be changes in the profile of fecal microbiomes, global fecal and urinary peroxidation markers, and neoplastic biomarkers.

**Discussion:**

Although epidemiological data support the promoting role of meat and the possible protective role of fish in colon carcinogenesis, no study has directly compared dietary profiles characterized by the presence of these two food groups and the role of the gut microbiome in these diet-associated CRC risks. This study will test the effect of these dietary profiles on validated CRC risk biomarkers.

**Trial registration:**

ClinicalTrials.gov, NCT03416777. Registered on 3 May 2018.

## Background

Colorectal cancer (CRC) is the second leading cause of cancer death in Europe. The geographical variation of incidence shows how environmental factors, particularly eating habits, play an important role in this disease [1]. Compelling evidence suggests that the risk of CRC is increased by consumption of red and processed meat as well as decreased by foods containing dietary fiber [2]. Although epidemiological studies suggest a reduction in the risk of CRC associated with fish consumption, the evidence for this link is still limited [2]. For non-starchy vegetables and fruits, although there is evidence indicative of a protective effect, this evidence is similarly considered limited and therefore less convincing than the promoting effect of red and processed meat [3].

Several hypotheses have been proposed to explain the association between red and processed meat and CRC: meat-based diets contain carcinogens formed during cooking but also lipid peroxidation and *N*-nitroso compounds whose formation is catalyzed by heme iron present in the colon following red and processed meat consumption [4]. Recent experimental studies [4, 5], epidemiological studies on the E3N cohort [6], and the randomized, double-blind, placebo-controlled SU.VI.MAX study [7] have demonstrated respectively the central role of heme iron and iron-induced peroxidation. On this basis, it has also been shown that antioxidants, in particular tocopherol, may help prevent colon cancer by decreasing the formation of mutagens arising from the peroxidation of fecal lipids, by decreasing oxidative stress in the epithelial cells of the colon, and by molecular mechanisms that influence cell death, cell cycle, and transcriptional events [6, 7]. Moreover, recent data have shown that consumption of a heme iron-enriched diet results in the alteration of gut microbiota consumption and function in rats that was associated with gut barrier defects, and that the gut microbiota is involved in heme-induced peroxidation [8]. On this basis, it has been reported that this microbiota is necessary for heme-induced hyperplasia and epithelial hyperplasia [9]. Despite these results, the role of the intestinal microbiome in determining the risk of cancer associated with red and processed meat has not yet been clarified.

The microbial fermentation of plant foods, associated with a low risk of CRC, increases intestinal short chain fatty acids (SCFAs), including butyrate, which has antineoplastic activity through its inhibition of histone deacetylase and the promotion of apoptosis [10], and also increases activated microbial phytochemicals, such as polyphenols with anti-inflammatory and antioxidant properties [11]. Consequently, it is known that diet influences the composition of the intestinal microbiome, as described by our group in a study on a rural human population [12], and the emerging evidence implies an involvement of the intestinal microbiota in CRC.

Cohort studies with subjects who consume various types of diets (e.g., omnivores, vegetarians, vegans) suggest that the diet alters the intestinal microbiome and the cytotoxic and genotoxic activities of the luminal colonic content [13]. However, although the association between the intestinal microbiota and the CRC is conceptually interesting, these results do not help to explain the mechanisms underlying the modulation of the intestinal microbiota by diet and the consequent impact on CRC risk.

We aimed to design this randomized, open, parallel clinical trial that will test whether diet effects on CRC risks are mediated by interaction with the intestinal microbiota and whether meat-based and pesco-vegetarian diets differentially modulate CRC risk biomarkers in clinically healthy subjects.

## Methods/design

### Study design

**Fig. 1:**
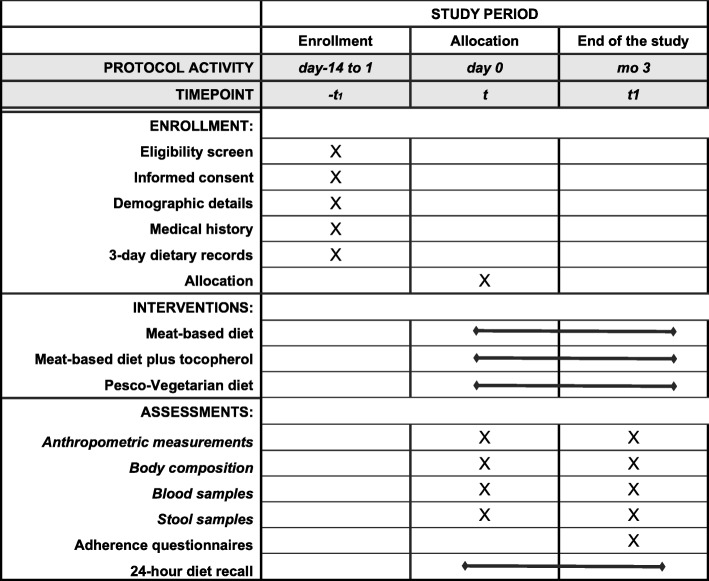
Time schedule of enrollment, interventions, and assessments for participants

The randomized, open, parallel clinical trial will be conducted at the Unit of Clinical Nutrition of the Careggi University Hospital in Florence, Italy. The study design follows the Standard Protocol Items: Recommendations for Interventional Trials (SPIRIT) guidelines (see Fig. 1 and Additional file 1).

### Eligibility criteria

Clinically healthy subjects (both sexes), aged 18–50 years, will be included in the study. The study population will be selected between these particular ages because after 50 years the risk of CRC shows a significant increase in incidence.

*Exclusion criteria* are the presence of current illness or unstable conditions that need medical supervision, current or recent (past 2 months) use of antibiotics or probiotics, pregnancy or intention to become pregnant in the next 12 months, lactation, smoking habit, and current or recent (past 2 months) adoption of vegetarian or vegan diets.

### Interventions and participant timeline

This clinical randomized study will use a parallel design with three intervention periods. After a 2-week run-in period, the eligible participants will be randomly assigned to three diets differing in the associated CRC risk: a meat-based diet (MBD, high risk), a nutritional control of a high-risk diet (MBD with alpha-tocopherol, MBD-T, presumably medium risk), and a pesco-vegetarian diet (PVD, low risk). Each diet period will last 3 months. The MBD will include 4 portions per week of red meat (1 portion = 150 g), 3 portions per week of processed meat (1 portion = 50 g), and 1 portion per week of poultry (1 portion = 150 g), for a total amount of 900 g per week of meat. The MBD-T with a nutritionally controlled risk will be similar to the MBD but supplemented with 100 mg/day of alpha-tocopherol. The PVD will exclude fresh and processed meat and poultry but will include 3 portions per week of fish, excluding shellfish (1 portion = 150 g), for a total amount of 450 g per week. The diets will contain other sources of proteins (e.g., eggs, dairy, legumes/beans), will be isocaloric, and will derive about 30% of energy from fats, 15% to 20% from proteins, and the rest from carbohydrates (mainly complex). No meals or supplements will be provided. Participants will prepare their meals or eat at restaurants. For all the diets, alcoholic beverages will be limited to one per day for women and two per day for men. Participants will be asked not to alter their physical exercise habits during the study.

The standardized baseline assessment for the intervention and control groups will include a questionnaire regarding demographic information, risk factors, and comorbidity. Furthermore, at the baseline visit, participants will be instructed on the objectives and methods of the clinical trial. Subjects who agree to participate in the study, after signing the informed consent, will be included and randomly divided into three groups, each assigned to consume the MBD, MBD-T, or PVD. Each participant will have to complete a 3-day dietary record (for 2 weekdays and a weekend day) before starting, and a dietician will analyze all 3-day dietary records using a country-specific food-nutrient database.

**Fig. 2:**
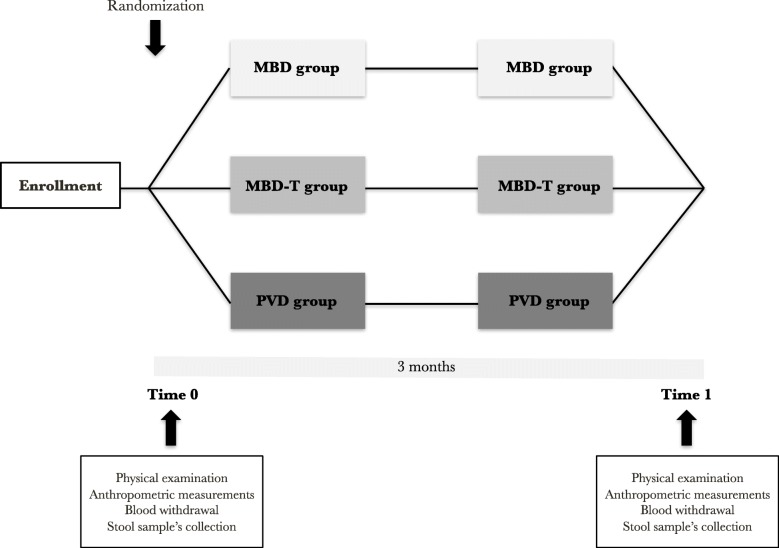
Organization of the intervention study

The study procedures are depicted in Fig. 2. There will be two clinical evaluations of the study population: at the beginning (Time 0) and at the end of the dietary interventions (Time 1).

### Outcome measures

#### Primary outcome

The primary outcome will be DNA damage (strand breaks and oxidized bases) in a human colon cancer cell line (HT29) exposed to fecal water. Genotoxic activity of fecal water will be determined by the comet assay, which evaluates the induction of DNA strand breaks and oxidized DNA bases upon exposure of the cells to fecal water [14]. The metric used will be changes in means from Time 0 to Time 1. Image acquisition will be performed with the Comet Assay IV software (Perceptive Instruments, UK). For each fecal water sample there will be a genotoxicity index (a single figure representing the mean area of the Comet tail [the percentage of DNA in the tail] measured in 100 cells). This figure will then be used in multivariate analyses to predict the potential operational taxonomy units (OTUs) measured using metagenomics approaches, potentially providing specific metabolic functions associated with the risk of neoplastic transformation [15, 16]. For this reason, this outcome was selected as the primary outcome of the study. In a previous study [14], fecal water activity markers were affected by specific dietary components linked with CRC risk such as red meat, and were sensitive to dietary supplementation with chemopreventive agents.

#### Secondary outcomes

Secondary outcomes will be measured in stool samples, blood, and urine. The metric used will be changes in means from Time 0 to Time 1. Regarding stool samples, we will evaluate the following:

- Fecal microbiota profile (fungi and bacteria) and fecal targeted metabolomic profile (SCFAs, amino acids, and secondary bile acids) changes from baseline. Total microbial DNA will be extracted from the participants’ faces using repeated bead-beating. The V3 and V4 hypervariable regions of the 16S rRNA gene for bacteria and the internal transcribed spacer (ITS1–4) for fungi will be sequenced on an Illumina MiSeq platform, following the Illumina protocol for 16S Metagenomic Sequencing Library Preparation. SCFAs, amino acids, and secondary bile acids will be measured by mass spectrometry (MS) on fecal samples. SCFAs will be analyzed using gas chromatography (GC)-MS, and bile acids by high-performance liquid chromatography (HPLC)–MS/MS analysis based on retention times and accurate masses of metabolites. The metabolites will be targeted and will be reported in absolute concentration. The amino acids will be analyzed by a combination of ultra high-performance liquid chromatography (UHPLC)-based hydrophilic interaction liquid chromatography (HILIC) and hybrid quadrupole time-of-flight (Q-Tof™) MS. The outcome measure will permit us to describe if and how the microbiome and the relative metabolomic profiles in clinically healthy subjects consuming three diets differing in their content of meat, meat products, and fish will change from baseline level. Numerous pathogenic microbes are known to promote CRC, but there is still limited evidence describing the impact of the diet on the composition of the fecal microbial populations and relative metabolites [17].
- Fecal water untargeted metabolomic profile changes from baseline. On selected samples, based on fecal water genotoxicity results, we will perform untargeted metabolomics by using one-dimensional (1H) nuclear magnetic resonance (NMR) spectroscopy. The outcome measure will permit us to analyze the qualitative change of fecal waters (bioavailable parts of fecal material) and potentially to identify new biomarkers associated with CRC risk.
- Mutagenicity and toxicity of fecal water changes from baseline [13, 14, 18]. The mutagenicity of fecal water will be tested using yeast as a model. The test will be performed in exponentially growing cells, because in these conditions cells are metabolically active. After the treatment, cell suspensions will be plated on complete media to detect viability and on two different selective media (without ilv or without trp) plates to evaluate survival and genetic effect.
- Markers of global peroxidation of fecal water (thiobarbituric acid reactive substances [TBARs]) and specific peroxidation of omega-3 and omega-6 polyunsaturated fatty acids (PUFAs) in fecal water changes from baseline [18, 19]. The level of global and specific peroxidation will be explored with the assay of respectively free 4-hydroxy hexenal (4-HHE) and free 4-hydroxy-2-nonenal (4-HNE). After derivatization, HHE and HNE determinations in fecal waters will be achieved by a sensitive and validated UHPLC-MS/MS multiple reaction monitoring (MRM) method using deuterated internal standards. Oxidative stress and inflammation are suspected to play a major role in the pathogenesis of CRC [20, 21].

In blood we will evaluate the following:

- Lipid profile change from baseline, including total cholesterol, low-density lipoprotein (LDL) cholesterol, high-density lipoprotein (HDL) cholesterol, and triglycerides
- Glycemic profile change from baseline
- Inflammatory profile change from baseline: high-sensitivity C-reactive protein (hs-CRP), circulating levels of inflammatory cytokines (interleukin-1ra [IL-1ra], interleukin-1 beta [IL-1β], IL-2, IL-4, IL-6, IL-8, IL-10, IL-12, IL-17, IL-18, tumor necrosis factor alpha [TNFα], interferon gamma [IFNγ], vascular endothelial growth factor [VEGF], monocyte chemoattractant protein-1 [MCP-1], and IFNγ-inducible protein 10 [IP-10]).

In urine we will analyze:

- 1,4-dihydroxynonane mercapturic acid (DHN-MA) change from baseline. This specific biomarker is the major urinary metabolite of HNE, a lipid peroxidation product [18].

### Sample size and power calculation

The sample size was determined to detect an anticipated change in fecal water-induced DNA damage evaluated through the comet assay of 25% from baseline, between the MBD and the PVD interventions. On the basis of a previously published trial conducted to test the efficacy of a dietary intervention on fecal water-induced DNA damage evaluated through the comet assay [14], a sample size of at least 50 in each group of the study will allow us to obtain a statistical power of 80% (beta) and alpha = 0.05, totaling 150 individuals. In case of loss to follow-up, extra volunteers will be enrolled to compensate. Losses will be included in the intention-to-treat but not in the per-protocol analyses.

### Recruitment and randomization

Male and female participants will be recruited using advertisements on local media, newspapers, social media, official papers, and websites. We will also recruit from our existing database of participants and friends or relatives of the hospital and university staff. After approval and completion of the initial assessment, the subjects will be formally included in the study and randomized with a simple 1:1:1 randomization to the intervention arms through a web-based online randomization procedure. No adaptive randomization procedures will be performed. The random allocation sequence will be produced and managed by an investigator who will not take part in the participants’ recruitment. The order of assignments will be kept hidden from the investigators who will enroll participants or assign interventions. Group allocation will be presented on a folded paper in a sealed envelope.

### Blinding

In this trial, blinding of participants and dieticians is not possible because there are obvious differences between the intervention diets. However, outcome measures in the present study cannot be easily influenced by the observer. Trial personnel who will enroll participants, data collectors, outcome assessors, and data analysts will be blinded to treatment allocation, and an employee outside of the research team will input data into the computer on separate data sheets. On the other hand, making the trial open rather than blinded may improve recruitment.

### Data collection

Follow-up assessments and data collection will be undertaken by trial personnel at the Unit of Clinical Nutrition of the Careggi University Hospital in Florence, Italy. All subjects will be examined between 6:30 and 9:30 a.m. after a 12-h fasting period.

#### Compliance

Compliance with the intervention is critical to the success of this project and will be achieved using behavior change strategies including self-monitoring, and regular phone calls for dietary counseling. To ensure good compliance, participants will be given a detailed 1-week menu plan for each dietary period with all foods expressed in weight and/or volume measurements. Participants will be contacted at least twice during the study period to promote diet compliance. A dietician will make unannounced telephone calls, and each participant will recall his or her 24-h diet. Participants may discontinue the intervention or withdraw from the study for the following reasons: (1) at the request of the participant; (2) if the investigator considers that a participant’s health will be compromised due to adverse events or a concomitant illness that develops after entering the study. Participants prematurely discontinued from the study before the 3-month evaluation will have the baseline clinical and laboratory evaluations performed.

#### Anthropometric measurements and body composition

Weight and height will be measured using a stadiometer. Body mass index (BMI) will be calculated as weight (kilograms)/height (square meters). Body composition will be determined with a bioelectrical impedance analyzer device (Akern, model SE 101) at the baseline and follow-up visits.

#### Blood samples

Blood samples will be collected at the beginning and at the end of each intervention phase. Blood samples will be centrifuged at 3000 rpm for 15 min, aliquoted to yield serum, and then stored at − 80 °C until analyses. The following biochemical measurements will be evaluated: complete blood count, lipid profile (total cholesterol, LDL cholesterol, HDL cholesterol, triglycerides), fasting glucose, liver function tests (aspartate aminotransferase, alanine transaminase, gamma-glutamyl transferase), renal function tests (serum creatinine, urea, uric acid), mineral profile (sodium, potassium, magnesium, calcium), iron metabolism (iron, ferritin), vitamin profile (vitamin B_12_, folic acid), pro- and anti-inflammatory profile (hs-CRP), and circulating levels of inflammatory cytokines (IL-1ra, IL-1β, IL-2, IL-4, IL-6, IL-8, IL-10, IL-12, IL-17, IL-18, TNFα, IFNγ, VEGF, MCP-1, and IP-10)*.* All the cytokines will be determined with the Bio-Plex cytokine assay (Bio-Rad Laboratories Inc., Hercules, CA, USA), according to the manufacturer’s instructions.

#### Stool samples

Stool samples (four or five scoops totaling 4 g) will be collected before and after each intervention phase. Stool sample collection kits, including containers, will be provided for the participant. The following stool measurements will be evaluated: fecal microbiota profiles (bacteria and fungi), targeted metabolomic profiles (SCFAs, amino acids, and secondary bile acids such as deoxycholic acid), genotoxicity and cytotoxicity of fecal water (on normal and preneoplastic colon epithelial cells), mutagenicity of fecal water (yeast model), markers of global peroxidation of fecal water (heme, TBARs), and markers of specific peroxidation of omega-3 and omega-6 PUFAs in fecal water.

#### Storage of biological specimens

The storage of biological specimens will be performed under appropriate conditions, according to standard methods. Blood samples will be aliquoted and stored at − 20 °C for 4 years before being used or destroyed. The stored samples will be used exclusively for research purposes upon consent of the donor. The destruction of samples will be appropriately documented.

### Data management

Data will be collected on an electronic database. Identifiable data or other documents will not be recorded in the database, and participants will be identified by a unique trial ID only. Hard copies of data sheets linking the participants’ ID numbers to their contact details will be kept securely in a locked filing cabinet in a locked office, accessible only to key research team members. Participant files and other source data (including copies of protocols, questionnaires, original reports of test results, correspondence, records of informed consent, and other documents pertaining to the conduct of the study) will be kept for the maximum period of time permitted by the institution. All of the project data will be stored in the Data Sharing In Nutrition (DASH-IN) infrastructure, which is developed by the European Nutritional Phenotype Assessment and Data Sharing Initiative (ENPADASI). Thereby we will adhere to the FAIR principles (Findable, Accessible, Interoperable, and Reusable). The data will be made open access upon publication. Within Europe the ELIXIR infrastructure is coordinating data stewardship and management activities in the life sciences.

Multiple strategies will be employed to improve data quality during data collection, including an accurate recruitment, a structured and time-limited protocol, the inclusion of a run-in period, the limitation of the burden and inconvenience of data collection on the participants, the development of a trusting and collaborative relationship between research units and participants, and double data entry.

### Statistical analysis

Outcomes will be analyzed by intention-to-treat and on-treatment procedures. The primary outcome (fecal water genotoxicity) will be analyzed within each group using paired comparison *t* tests to evaluate whether the changes from baseline to 3 months will be statistically significant. The absolute change (mean value at baseline subtracted from the mean value after intervention for each subject) will be estimated with independent *t* sample tests. One-way analysis of variance will be used for testing differences between changes in the three diet groups. A general linear model for repeated measurements (after checking regression assumptions) will be performed in order to compare the effects of the three different treatments. A model with adjustments for multiple confounders will be used. Data for the general linear model will be reported as geometric means with their standard errors. Multiple strategies will be employed to reduce attrition, including an accurate recruitment, a structured and time-limited protocol, the inclusion of a run-in period, the limitation of the burden and inconvenience of data collection on the participants, and the development of a trusting and collaborative relationship between research units and participants. If a participant does not attend a scheduled appointment, a maximum of three telephone calls will be made and one email sent prior to withdrawing the participant from the study. If a participant wants to withdraw from the study intervention, the reason for withdrawal will be documented in the participant records for the subsequent analysis in the interpretation of the results.

Before starting the data analysis, the level, pattern, and likely causes of the missingness in the baseline variables and outcomes will be investigated by forming appropriate tables. This information will be used to determine whether the level and type of missing data have the potential to introduce bias into the analysis results or substantially reduce the precision of estimates for the proposed statistical methods. Sensitivity analyses will be undertaken, based on the assumption that missing outcomes are the worst possible, or the best possible, in different randomization groups. If these analyses show that conclusions may differ based on missing values, then supplementary multiple imputation for missing values will be undertaken. These analyses will account for results of any losses to follow-up insofar as they pertain to differences in measured variables (i.e., under the assumption of missing at random). A *p* value < 0.05 will be considered statistically significant. Statistical analyses will be performed using SPSS software for Macintosh (SPSS Inc., Chicago, IL, USA).

### Monitoring

Given the limited objectives and its short-term nature, this trial will be monitored on a regular basis by the protocol team and the local Institutional Review Board, without the use of a formal data monitoring committee. Each month, the protocol team will provide the local Institutional Review Board with a monitoring report, including a review of activities, progress, difficulties, and issues of concern. No interim analysis will be performed. Data access will be restricted to trained staff with unique password-protected accounts. Adverse events such as unfavorable and unintended signs, abnormal laboratory findings, symptoms, or diseases temporally associated with the intervention diet will be collected from the time of randomization until the final 3 months follow-up visit for each participant, whether or not considered related to the intervention study. All adverse events will be followed up until they are resolved.

## Discussion

The Western diet typically based on red and processed meat is considered a risk factor in colon carcinogenesis [2]. However, although various mechanisms have been implicated in causing this risk, it is not yet clear whether the microbiome has a role in this process [22]. Concurrently, increasing evidence has been reported in the literature on the beneficial role of fish consumption on the pathogenesis of CRC, but without convincing results [2]. In recent years, the number of subjects who have begun to adopt a plant-based dietary pattern has increased compared to the past, when the vegetarian population was limited to only a few selected cohorts. This increase is mainly attributed to ethical and environmental motivations, as well as to health concerns.

To date, no studies are available that evaluate the effects on colon carcinogenesis of a meat-based diet and a diet that excludes meat and meat derivatives but includes fish. The aim of the project will be to understand the role of the intestinal microbiota as a determinant of the effect of diet on CRC risk and to identify specific microbiota/metabolomic profiles associated with cancer risk. The comparison between meat-based and pesco-vegetarian dietary models in terms of cancer risk prevention will be of fundamental clinical importance for the general population and will contribute to a better understanding of the possible metabolic mechanisms underlying the health consequences associated with adherence to both diets in improving the neoplastic risk profile of clinically healthy subjects.

### Trial status

The trial has received all necessary regulatory approvals. The current approved protocol version is 2.0 (version date March 1, 2018). We anticipate a September 15, 2019 start date for recruitment and a September 15, 2020 recruitment completion date.

## Supplementary information

**Additional file 1.** SPIRIT 2013 checklist: recommended items to address in a clinical trial protocol and related documents.

## Data Availability

The results from this clinical trial have the potential for immediate public health applicability for colon cancer prevention. The target audience will be reached through publications, oral presentations, and seminars. Data analysis and manuscript preparation will occur during the last 6 months of this proposed trial. All plans for dissemination of study results will be discussed with the investigators before implementation. Any amendments to the protocol and information provided to participants will be submitted to the Ethics Committee for approval prior to implementation. Substantial amendments may only be implemented after written approval has been obtained, whereas non-substantial amendments can be implemented without written approval from the Ethics Committee. The Chief Investigator must ensure that the participant’s privacy is maintained. Data and source documents will be stored in such a way that they can be accessed at a later date for purposes of monitoring or inspection by the Ethics Committee. At the end of the study, participants will be able to request a copy of the results of the study from the Chief Investigator. The results from the trial will be submitted for publication in a peer-reviewed journal irrespective of the outcome. The final report will follow the Consolidated Standards of Reporting Trials (CONSORT) 2010 guidelines. Authorship of presentations and reports related to the study will be in the name of the collaborative group.

## Abbreviations

BMI: Body mass index
CRC: Colorectal cancer
HbA1c: Glycated hemoglobin
HDL: High-density lipoprotein
hs-CRP: High-sensitivity C-reactive protein
IFNγ: Interferon gamma
IL-10: Interleukin-10
IL-12: Interleukin-12
IL-17: Interleukin-17
IL-18: Interleukin-18
IL-1ra: Interleukin-1ra
IL-1β: Interleukin-1 beta
IL-2: Interleukin-2
IL-4: Interleukin-4
IL-6: Interleukin-6
IL-8: Interleukin-8
IP-10: IFNγ-inducible protein 10
LDL: Low-density lipoprotein
MCP-1: Monocyte chemoattractant protein-1
TNFα: Tumor necrosis factor alpha
VEGF: Vascular endothelial growth factor
